# Council tax valuation band predicts breast feeding and socio-economic status in the ALSPAC study population

**DOI:** 10.1186/1471-2458-6-5

**Published:** 2006-01-11

**Authors:** Norman Beale, Gill Kane, Mark Gwynne, Carole Peart, Gordon Taylor, David Herrick, Andy Boyd, ALSPAC Study Team

**Affiliations:** 1 Northlands R & D General Practice, Calne, SN11 0HH, UK; 2 Bath & Swindon RDSU, Wolfson Centre, Royal United Hospital, Bath BA1 3NG, UK; 3 Unit of Paediatric and Perinatal Epidemiology, Department of Community Based Medicine, University of Bristol, 24 Tyndall Avenue, Bristol BS8 1TQ, UK

## Abstract

**Background:**

Breast-feeding rates in the UK are known to vary by maternal socio-economic status but the latter function is imperfectly defined. We test if CTVB (Council Tax Valuation Band – a categorical assessment of UK property values and amenities governing local tax levies) of maternal address predicts, in a large UK regional sample of births, (a) breast-feeding (b) personal and socio-economic attributes of the mothers.

**Methods:**

Retrospective study of a subset (n.1390 selected at random) of the ALSPAC sample (Avon Longitudinal Study of Parents and Children), a large, geographically defined cohort of mothers followed from early pregnancy to 8 weeks post-delivery. Outcome measures are attitudes to breast-feeding prior to delivery, breast-feeding intention and uptake, demographic and socio-economic attributes of the mothers, CTVB of maternal home address at the time of each birth. Logistic regression analysis, categorical tests.

**Results:**

Study sample: 1360 women divided across the CTVBs – at least 155 in any band or band aggregation. CTVB predicted only one belief or attitude – that bottle-feeding was more convenient for the mother. However only 31% of 'CTVB A infants' are fully breast fed at 4 weeks of life whereas for 'CTVB E+ infants' the rate is 57%. CTVB is also strongly associated with maternal social class, home conditions, parental educational attainment, family income and smoking habit.

**Conclusion:**

CTVB predicts breast-feeding rates and links them with social circumstances. CTVB could be used as the basis for accurate resource allocation for community paediatric services: UK breast-feeding rates are low and merit targeted promotion.

## Background

When the UK National Health Service began some 50 years ago, over 75% of British mothers initiated breast-feeding in their infants [1]. By 1970, a generation later, there had been a marked decline to something like half the former rate [2]. But a new phenomenon had appeared. There was now a significant social divide in how mothers preferred to feed their infants: 60% of Registrar General Social Class I women fed their babies at the breast but the rate in Social Class V women had plummeted to 24% [2]. Although breast-feeding has regained some popularity in the UK, the social divide remains. As recently as 1995, the National Infant Feeding Survey [3] showed that whereas 89% of women who had gone on to tertiary education before motherhood breast-fed their infants, only 52% of their counterparts who had left school at 16 did so. This spectrum is corroborated by a more recent study emanating from UK General Practice [4] and has very important implications for infant health. Breast-fed babies are clearly less prone to gastrointestinal tract infections and eczema [5] and, in some studies, to excessive weight gain, upper respiratory infections and even sudden death [6]. Moreover, there is now good evidence that breast-feeding is fundamentally important for long-term health: breast milk contains unique developmental promoters and is free of inappropriate dietary antigens such as bovine protein [7].

Studies of how breast-feeding relates to socio-economic status in the UK are beset by the inherent problems always found in allotting social and economic status correctly. Familiar proxy markers of socio-economic status such as home-ownership or years of education can misattribute. So, also, do compound indices such as that of Townsend [8] or Carstairs [9] that are also bedevilled by the inevitable fallacies of area statistics based on aggregated Census data [10]. Our so-called 'knowledge' of how social factors affect clinical phenomena such as infant feeding is more than a little uncertain [11] and deserves improvement. There is also the issue of how cigarette smoking influences breast-feeding, a complex consideration since smoking frequency, itself, has come to be unevenly distributed among the social classes [12].

The Avon Longitudinal Study of Parents and Children (ALSPAC) was initiated in 1990. In order to be eligible, women had to be pregnant with expected dates of delivery between 1/4/91 and 31/12/92 and living in Avon, UK. The core sample consists of 14,541 pregnancies, of which 13,868 resulted in at least one live born child. Data have been collected regularly on the mothers and their offspring since recruitment [13]. It is the largest longitudinal study of childhood in the world, aiming to reveal how physical and social environment interact, with genetic inheritance, to affect all aspects of a child's health and development. It is already seen as a very valuable database (well over 200 papers published so far) and this new study links information from the antenatal and perinatal questionnaires to a novel marker of socio-economic standing at household level.

In 1992 the British Government replaced the Community Charge ('Poll Tax') with a new tax – the Council Tax [14]. Homes were to be allotted an 'open market' value, as at 1 April 1991, based on size, layout, character and locality, and placed into one of eight 'Valuation Bands'. The bands were so structured that the most modest homes – estimated value then less than £40,000 – were placed in band A, the next group – between £40,000 and £52,000 in band B and so on progressively up to the most expensive homes – values exceeding £320,000 – in band H. The allotted bands, A – H, then dictate the amount of the annual tax. All UK Local Authorities were mandated to levy the new tax and to publish lists showing the Council Tax Valuation Band (CTVB) of all properties in their jurisdiction: these are now available, for England and Wales, on a web site published by the Valuation Office Agency [15]. We first examined this new 'ecological attribute' of all patients, the CTVB of their residence, in a small study reported in 2000 [16]. We demonstrated an association between CTVB and (a) established socio-economic indicators viz. home ownership and car access and (b) clinical demand in a typical UK general practice. We have also reported that CTVB is a significant predictor of mortality [17] and of face-to-face consultation rates [18] and overall workload in general practice [19]. These studies are the only reports in the literature that use CTVB as a marker linking clinical parameters to the socio-economic status of patients.

In this study we aim to test the hypothesis that CTVB of the home address of new mothers is a predictor of breast-feeding intention and establishment. We test, further, whether CTVB is a marker of close environmental influences on maternal health such as smoking habit and housing quality. In so doing, we also aim to illuminate (a) that CTVB is a marker of socio-economic status and (b) that breast-feeding is governed socially. There are no reports of any previous studies that so utilise CTVB.

## Methods

We investigated a random anonymised sample of the responses made, by ALSPAC participants [13], to serial questionnaires administered during, and at 4 – 8 weeks after, their pregnancies. Our anonymised sample was obtained by selecting 2000 pregnancies, at random, from all eligible pregnancies and a CTVB was obtained, for each, from their recorded contemporaneous addresses via the Valuation Office website [15]. The sample was then subdivided by CTVBs and analysed in respect to categorical responses to questions on:

(A) Beliefs and intentions about infant feeding – administered during their pregnancies:

1. 'bottle feeding is more convenient for mum'

2. 'breast feeding is difficult'

3. 'breast feeding restricts mum's freedom'

4. 'breast feeding leads to a special bond'

5. intention to breast feed in first week of life

6. intention to breast feed in next three months of life.

(B) feeding outcome – administered four weeks after confinement:

1. any breast-feeding at all

2. fully breast feeding on first day of life

3. fully breast feeding 2^nd ^– 6^th ^days of life

4. fully breast feeding 2^nd ^week of life

5. fully breast feeding 3 week of life

6. fully breast feeding 4^th ^week of life

(C) personal circumstances – administered during ^a ^and after^b ^their pregnancies:

1. mother's age at birth of the child^a^

2. mother's present marital status^a^

3. no. other children with mo/partner as natural parents^a^

4. mother smoking cigarettes during pregnancy^a^

5. mother smoking cigarettes regularly since birth of child^b^

(D) circumstances of their confinement – from medical records^c^, maternal report 8 weeks after confinement^d^:

1. where baby born^d^

2. analgesia used in labour^d^

3. had caesarian section^d^

4. had pre-term delivery (24 – 36 weeks)^c^

5. had multiple pregnancy^c^

6. baby admitted to scbu^d^

7. had a boy^c^

8. birth weight^c^

(E) socio-economic circumstances – administered during^a ^and after^b ^their pregnancies:

1. mother's social class by Registrar General classification^a^

2. mother's family income at recruitment^b^

3. mother receiving money from DHSS for baby equipment^b^

4. mother's highest educational attainment^a^

5. partner's highest educational attainment^a^

6. mother lives in owner-occupied or rented accommodation^a^

7. number of living rooms (excl. kitchen) in mother's accommodation^a^

8. mould or damp in the mother's home^a^

### Statistical method

Logistic regression, incorporating forward, step-wise, conditional selection, was used with outcome measure being 'fully breast-feeding at four weeks of life' and applying all other variables from sections C, D & E above. Forward, step-wise, conditional selection showed that (a) CTVB and (b) mother's highest educational attainment were both independent and significant markers of outcome measure. Analyses were performed using SPSS version 11.0.1.

### Ethical consent

All data collection was discussed in detail with the ALSPAC Ethics and Law Advisory Committee as well as being approved by the local LRECs.

## Results

Of the 2000 pregnancies investigated for CTVB, 1390 were in the core ALSPAC sample of 14,541. 17 twin pregnancies were excluded and this resulted in a 70% sample – 1373 singleton pregnancies, of which 1318 resulted in a live birth. The youngest mother was 15 years, the oldest 44 years. Median age was 27 years; inter-quartile range 24 – 31 years. It was possible to allocate a CTVB to 1360 of the quoted home addresses – a 99% attribution rate. The numbers and proportions of mothers in each CTVB are shown in Table 1. CTVBs E -H inclusive were aggregated for analysis because of small numbers.

The findings for section A – beliefs and intentions about breast-feeding – are shown in Table 2: differences are not significant, with the exception of perceived 'bottle-feeding convenience'. However, there are consistently significant differences in breast-feeding intention.

Feeding outcomes are shown in Table 3: there is a consistent and highly significant trend towards women living in higher CTVB breast-feeding their babies. Progressive conversion away from exclusive breast-feeding is parallel in all CTVBs. These findings appear to demonstrate that CTVB predicts breast-feeding but multivariate testing is needed to ensure they are not confounded by any of the personal factors listed in section C, nor by any of the perinatal circumstances listed in D. In fact, forward, step-wise, conditional selection showed that (i) mother's educational attainment and (ii) CTVB were both independent and significant markers of the outcome measure (infants being fully breast-fed at four weeks).

Associations were then tested between CTVB and the range of socio-economic variables in section E. Table 4 shows how CTVB consistently and significantly predicts these markers and, also, smoking habit (from section C).

## Discussion

Our analyses show that both mother's educational attainment and CTVB predict breast-feeding establishment. CTVB is, we suggest, the more useful marker since it can be obtained independently of the subjects concerned and, besides being more convenient and accessible, is therefore more objective. CTVB is also a novel finding, remarkable in the consistency with which it predicts the successive phases of breast-feeding in different groups of a cohort of UK mothers (Figure1). Moreover it predicts, with equal symmetry, the maternal socio-demographic circumstances. Linking these two paradigms then shows how breast-feeding is socially governed in the UK. 80% of new babies taken home to large, mostly detached and owner-occupied homes (CTVB 'E+') were breast-fed. Of their counterparts in modest homes – mostly rented rooms, flats or conjoined houses (CTVB 'A') – only some 50% were breast-fed and it was seven times more likely that their mothers would be smokers. The latter young families also had significantly less weekly income, had had less education, and were more likely to have needed the subsidised provision of baby equipment by the DSS. Superficially, much of this is hardly new information and, therefore, no surprise. But the pioneering use of CTVB as the demographic marker in the study is unique: it gives us unequivocal categorisation, objectivity and multiple insights. The study data – from 1991/2 – may be challenged as somewhat out of date but they can certainly be taken as representative, participants being an adequate and randomised sub-set of the 14,541 ALSPAC (13) responders, mothers then confined in Avon.

The main implications of the study are probably threefold:

I. for the distribution and health promotion of breast-feeding and the implicit resource allocation determinants;

II. for the association of breast-feeding and cigarette smoking;

III. for the prospects of CTVB as a valid socio-economic marker.

I. The social mal-distribution of good feeding practice, viz. breast-feeding during the initial months of life, carries serious implications for health promotion. With the exception of seeing bottle-feeding as more convenient (implications being less 'tied' to the baby and never having to breast-feed in public), early beliefs and attitudes to infant feeding are equivalent in all the women. Despite this, far fewer mothers from less prosperous homes eventually put their newborns to the breast. This discrepancy deserves further examination: there may be clues that could help clinicians raise breast-feeding rates in these women and, therefore, overall. But enlightened or not, more sensitive and more determined counselling is obviously necessary where it is most needed – among the socio-economically deprived. These can now be spotted, prospectively and simply, by referring to the CTVB of their addresses at antenatal booking. The implications for resource allocation to UK general practice and other childcare facilities in the community, for optimum manpower dispersal, are obvious and reinforce previous conclusions [20]. And, as a rider, our findings also suggest that local or regional claims for 'success' in achieving high breast-feeding rates should be disputed until they have been modulated by social class distribution in the catchment population.

II. The inverse association between breast-feeding and smoking in young mothers is well known. There is a consensus that the link is a social rather than a physiological one [21] for although there is evidence that smoking diminishes hypothalamic activity and, therefore, potential milk production and flow [22], the strength of this inhibition is contentious and probably marginal. Our findings reinforce the dominance of the social mechanism. Among the numerous factors tested, smoking included, CTVB is the strongest predictor for breast-feeding. Being a smoker neither deters nor determines infant feeding habit: of our 211 mothers who smoked in the last two months of pregnancy (18.5%), there is still a progressive diminution of breast-feeders from CTVB 'E+' women to their 'A' counterparts.

III. The unifying and innovatory aspect of this study is the use of CTVB in grouping study subjects for cross-sectional comparison. The proposal that in so doing we are placing women in a socio-economic spectrum is supported by our previous publications [16,23] and reinforced, strongly, by the subsidiary findings of the study itself. But whether CTVB is, or is not, a satisfactory socio-economic surrogate, it certainly has some inherent strengths to recommend it. It is an official and categorical device instituted and maintained by a body (The Government) outside the debate and can therefore be considered objective. It is also universal, comprehensive and stable. The bands need revision but this is scheduled for England and has already occurred in Wales [24]. CTVB is also readily available, on-line [15], for every property in the UK and, since it is an individual household attribute, is entirely free of the perils of Census data interpretation [10] and of the so-called 'ecological fallacy' [25] inherent in gleaning aggregated data from within a variety of geographical boundaries. And unlike educational attainment, which this study also shows to be a marker of breast-feeding, CTVB can be obtained without access to, and enquiry of, the individuals concerned. This makes it easier to obtain on a massive scale if need be and CTVB certainly meets the essential criteria for a valid socio-economic marker as specified by Wagstaff and colleagues in 1991 [26]: it reflects inequality, does so across the whole population, and is sensitive to changes in that population. In fact CTVB could have a significant future as an epidemiological tool: many other studies suggest themselves.

## Conclusion

CTVB predicts breast-feeding establishment and could be an objective and accessible marker since it can be obtained easily and independently of the subjects concerned. It also predicts, with equal symmetry, maternal socio-demographic circumstances and therefore demonstrates how breast-feeding is socially governed in the UK.

## Competing interests

The author(s) declare that they have no competing interests.

## Authors' contributions

NB and GK conceived the study and performed a pilot project; DH and AB provided the ALSPAC data; CP and MG matched the data to Council Tax Bands; NB and GT analysed the data; NB wrote the manuscript, the final version of which was read and approved by all authors.

## Pre-publication history

The pre-publication history for this paper can be accessed here:



## Figures and Tables

**Table 1:** numbers of study mothers and their distribution across the CTVBs – bands E and above aggregated to obviate small numbers.

	Number of mothers	Percentage of total
CTVB		
A	256	18.6
B	494	36.0
C	299	21.8
D	156	11.4
E+	155	11.3
Unknown	13	1.0
Total	1373	100

**Table 2:** beliefs and intentions of the study mothers (n. 1360) during pregnancy.

	**beliefs**								**intention to feed**			
	**bottle-feeding more convenient for mum**		**breast-feeding is more difficult**		**breast-feeding restricts mum's freedom**		**breast-feeding leads to special bond**		**in first week**		**in first three months of life**	

	**strongly agree, agree (percent)**	**other (1)**	**strongly agree, agree (percent)**	**other (1)**	**strongly agree, agree (percent)**	**other (1)**	**strongly agree, agree (percent)**	**other (1)**	**breast only (percent)**	**other (1)**	**breast only (percent)**	**other (1)**

**CTVB**												
**A**	104 (54)	90	57 (30)	136	43 (22)	150	151 (78)	42	121 (64)	68	62 (33)	126
**B**	177 (44)	224	103 (25)	304	87 (21)	320	329 (81)	78	285 (71)	117	155 (39)	244
**C**	110 (41)	156	66 (25)	200	54 (20)	212	218 (82)	48	204 (78)	59	121 (46)	140
**D**	45 (32)	96	37 (26)	104	16 (11)	125	118 (84)	23	122 (88)	16	70 (50)	70
**E+**	38 (29)	95	31 (23)	102	29 (22)	104	116 (87)	17	119 (91)	12	72 (55)	60
												
	474	661	294	846	229	911	932	208	851	272	480	640
**Total**	1135		1140		1140		1140		1123		1120	
	X^2 ^for trend = 26.04		X^2 ^for trend = 1.03		X^2 ^for trend = 1.67		X^2 ^for trend = 4.65		X^2 ^for trend = 47.09		X^2 ^for trend = 21.25	
	P < 0.001 (1 d.f.)		P = 0.31 (1 d.f.)		P = 0.197 (1 d.f.)		P = 0.031 (1 d.f.)		P < 0.001 (1 d.f)		P < 0.001 (1 d.f)	

(1) not all women responded to all questions on questionnaires

**Table 3:** feeding outcomes of the study mothers (n. 1360) after confinements.

	**feeding outcomes**											
	**any breast-feeding at all**		**fully breast-fed on first day of life**		**fully breast-fed on days 2 – 6**		**fully breast-fed second week of life**		**fully breast-fed third week of life**		**fully breast-fed fourth week of life**	

	**breast-fed (percent)**	**other (1)**	**breast-fed (percent)**	**other (1)**	**breast-fed (percent)**	**other (1)**	**breast-fed (percent)**	**other (1)**	**breast-fed (percent)**	**other (1)**	**breast-fed (percent)**	**other (1)**

**CTVB**												
**A**	120 (62)	73	98 (54)	83	97 (53)	85	83 (46)	99	69 (40)	113	58 (31)	129
**B**	311 (72)	118	260 (64)	249	243 (59)	169	226 (54)	189	178 (43)	237	153 (36)	267
**C**	226 (82)	51	194 (72)	76	168 (62)	101	163 (60)	109	132 (49)	139	117 (43)	157
**D**	130 (88)	18	111 (77)	33	108 (76)	35	104 (73)	39	89 (63)	53	81 (57)	62
**E+**	65 (89)	8	117 (85)	20	109 (80)	27	96 (70)	41	90 (65)	49	80 (57)	60
												
	852	268	780	361	725	417	672	477	558	591	489	675
**Total**	1120		1141		1142		1149		1149		1164	
	X^2 ^for trend = 43.5		X^2 ^for trend = 73.40		X^2 ^for trend = 35.14		X^2 ^for trend = 32.04		X^2 ^for trend = 37.52		X^2 ^for trend = 32.25	
	P < 0.001 (1 d.f)		P < 0.001 (1 d.f)		P < 0.001 (1 d.f)		P < 0.001 (1 d.f)		P < 0.001 (1 d.f)		P < 0.001 (1 d.f)	

1) not all women responded to all questions on questionnaires

**Table 4:** personal and socio-economic factors of the study mothers tested versus CTVB of home address (n.1360).

**Personal or socio-economic factor**	**Range cross-tabulated versus CTVBs A – E+**	**X^2 ^for trend**	**Degrees of freedom**	**P value**
**Mother's social class by RG classification**	I, II, IIINM, IIIM, IV, V	95.30	1	< 0.001
**Mother's weekly family income at recruitment**	< £100, £100–199, £200–299, £300–399, >£400	128.6	1	< 0.001
**Money from DSS for baby equipment**	Yes or no	30.3	1	< 0.001
**Mother's highest educational attainment**	CSE, Vocational, O-level, A-level, Degree	136.0	1	< 0.001
**Partner's highest educational attainment**	CSE, Vocational, O-level, A-level, Degree	101.9	1	< 0.001
**Mother's accommodation status**	Owned, mortgaged, council-rented, housing association rented, privately rented (furnished), privately rented (unfurnished), other	73.7	1	<0.001
**Number living rooms in mother's accommodation**	0 – 10 inclusive	126.0	1	< 0.001
**Mould or damp in mother's accommodation**	Yes or no	10.0	1	0.002
**Mother smoking cigarettes in pregnancy**	Yes or no	73.6	1	< 0.001
**Mother smoking cigarettes since birth of child**	Yes or no	75.0	1	< 0.001
